# Pre-Operative Prediction of Mediastinal Node Metastasis Using Radiomics Model Based on ^18^F-FDG PET/CT of the Primary Tumor in Non-Small Cell Lung Cancer Patients

**DOI:** 10.3389/fmed.2021.673876

**Published:** 2021-06-18

**Authors:** Kai Zheng, Xinrong Wang, Chengzhi Jiang, Yongxiang Tang, Zhihui Fang, Jiale Hou, Zehua Zhu, Shuo Hu

**Affiliations:** ^1^Department of Nuclear Medicine, Xiangya Hospital, Central South University, Changsha, China; ^2^Positron Emission Tomography/Computed Tomography (PET/CT) Center, Hunan Cancer Hospital, Changsha, China; ^3^The Affiliated Cancer Hospital of Xiangya School of Medicine, Central South University, Changsha, China; ^4^General Electric (GE) Healthcare (China), Shanghai, China; ^5^National Clinical Research Center for Geriatric Disorders, Xiangya Hospital, Central South University, Changsha, China; ^6^Key Laboratory of Biological Nanotechnology of National Health Commission, Xiangya Hospital, Central South University, Changsha, China

**Keywords:** non-small cell lung cancer, ^18^F-FDG PET/CT, radiomics analysis, lymph node staging, predict, primary tumor

## Abstract

**Purpose:** We investigated whether a fluorine-18-fluorodeoxy glucose positron emission tomography/computed tomography (^18^F-FDG PET/CT)-based radiomics model (RM) could predict the pathological mediastinal lymph node staging (pN staging) in patients with non-small cell lung cancer (NSCLC) undergoing surgery.

**Methods:** A total of 716 patients with a clinicopathological diagnosis of NSCLC were included in this retrospective study. The prediction model was developed in a training cohort that consisted of 501 patients. Radiomics features were extracted from the ^18^F-FDG PET/CT of the primary tumor. Support vector machine and extremely randomized trees were used to build the RM. Internal validation was assessed. An independent testing cohort contained the remaining 215 patients. The performances of the RM and clinical node staging (cN staging) in predicting pN staging (pN0 vs. pN1 and N2) were compared for each cohort. The area under the curve (AUC) of the receiver operating characteristic curve was applied to assess the model's performance.

**Results:** The AUC of the RM [0.81 (95% CI, 0.771–0.848); sensitivity: 0.794; specificity: 0.704] for the predictive performance of pN1 and N2 was significantly better than that of cN in the training cohort [0.685 (95% CI, 0.644–0.728); sensitivity: 0.804; specificity: 0.568], (*P*-value = 8.29e-07, as assessed by the Delong test). In the testing cohort, the AUC of the RM [0.766 (95% CI, 0.702–0.830); sensitivity: 0.688; specificity: 0.704] was also significantly higher than that of cN [0.685 (95% CI, 0.619–0.747); sensitivity: 0.799; specificity: 0.568], (*P* = 0.0371, Delong test).

**Conclusions:** The RM based on ^18^F-FDG PET/CT has a potential for the pN staging in patients with NSCLC, suggesting that therapeutic planning could be tailored according to the predictions.

## Introduction

Among all cancers, lung cancer remains the most commonly diagnosed (11.6% of the total cases) and leading cause of cancer death (18.4% of the total cancer deaths). Non-small cell lung cancer (NSCLC) accounts for 85% of the cases (1, 2). For patients newly diagnosed with NSCLC, the exact evaluation of the pathological lymph node (LN) status plays an important role in the choice of therapy regimen. There is a consensus that lobectomy combined with systemic nodal dissection is the recommended surgical treatment for early-stage NSCLC; however, sublobar resection and stereotactic body radiation therapy (SBRT) are possible alternatives for patients who are ineligible for lobectomy (3–5). Thus, accurate differentiation of pathological node-negative from positive is critical for selecting the optimal therapeutic plan.

Currently, one of the most widespread modalities used for the clinical LN (cN) staging of patients with NSCLC is fluorine-18-fluorodeoxyglucose positron emission tomography/computed tomography (^18^F-FDG PET/CT) (6, 7). Unfortunately, the accuracy of ^18^F-FDG PET for the direct evaluation of each mediastinal LN for the presence of metastasis is inherently limited by an avid FDG uptake that can be caused by inflammation due to infectious or non-infectious etiologies such as tuberculosis, pneumoconiosis, or chronic obstructive pulmonary disease (8–10). To improve the diagnostic ability of false-positive signs, several studies have analyzed the differences in parameters such as morphology, density, metabolism, and radiomics between benign and malignant LNs (11, 12). Nevertheless, because of the low FDG uptake, occult LN metastasis (OLM) in patients with NSCLC fails to be detected by ^18^F-FDG PET (13) and, hence, imaging is prone to false-negative signs. Accordingly, some researchers have tried to predict OLM by analyzing the ^18^F-FDG metabolic parameters of the primary tumor in NSCLC (14, 15). To the best of our knowledge, few researchers have dealt with both problems of false-positive signs and OLM in mediastinal LN staging. Furthermore, few studies have investigated whether radiomics features derived from the primary lesion of NSCLC might provide useful information for mediastinal LN staging.

Therefore, we constructed and validated a radiomics model (RM) to predict the pathological mediastinal LN staging (pN0 vs. pN1 and pN2) based on the ^18^F-FDG PET/CT imaging of NSCLC primary tumors.

## Materials and Methods

### Patients

This study involving human participants was reviewed and approved by the Ethical Commission of Medical Research Involving Human Subjects at the Region of Xiangya Hospital, Central South University, China, and the requirement for informed consent was waived. We reviewed the electronic medical records of 716 consecutive patients with NSCLC [adenocarcinoma (ADC) and squamous cell carcinoma (SCC)] who underwent both ^18^F-FDG PET/CT staging and surgical resection with a curative intent from February 2007 to November 2019. All the patients underwent surgical resection with systematic mediastinal (N2) and hilar (N1) LN dissections within 2 weeks of ^18^F-FDG PET/CT examination. Pre-operative cN staging and post-operative pN staging of the patients were performed and recorded according to the eighth edition of the Union for International Cancer Control TNM classification (16). Histological types were diagnosed according to the World Health Organization classification. We excluded patients from the study if they had (i) histology other than ADC or SCC, (ii) history of other cancer, (iii) received any treatment before ^18^F-FDG PET/CT, and (iv) undergone pre-operative lung biopsy.

### ^18^F-FDG PET/CT Acquisition and Reconstruction

All ^18^F-FDG PET/CT scans were performed on a dedicated PET/CT scanner (Discovery ST8, GE Healthcare, Chicago, IL). All patients fasted for at least 6 h before imaging, and a blood glucose level of <110 mg/dL was confirmed before the administration of ^18^F-FDG. PET/CT was performed ~60 min after the intravenous injection of 370 MBq/kg of ^18^F-FDG. First, a low-dose CT scan without contrast enhancement (120 mA, 150 kV, 512 × 512 matrix, the pitch of 1.75, reconstruction thickness and interval of 3.75 mm) for a precise anatomical localization and attenuation correction was performed. Next, a three-dimensional PET scan (thickness of 3.27 mm) was performed from the skull base to the proximal thighs with an acquisition time of 3 min per bed position.

The PET data sets were iteratively reconstructed using an ordered-subset expectation maximization (OSEM) algorithm with attenuation correction. All collected images were displayed on the GE Healthcare Xeleris 3.0 to reconstruct the PET, CT, and PET/CT fusion images.

### Image Interpretation and Lesion Segment

Two experienced nuclear medicine physicians who were blinded to the patient's clinical information retrospectively reviewed the ^18^F-FDG PET/CT scans. Any difference of opinion was resolved by consensus. Mediastinal and hilar LNs with a short axis of ≥10 mm in the short axis on CT and with a high accumulation of ^18^F-FDG compared with that of the adjacent mediastinal tissue were considered as cN2 or cN1 at our institution. Fused PET/CT images were viewed on the Advantage Workstation (version AW 4.7, GE Healthcare).

The region of interest (ROI) for each patient was delineated initially around the tumor outline for the largest cross-sectional area of the primary lung lesion on both the CT and PET images. The ROIs were segmented manually by a single experienced nuclear medicine physician, and the final ROIs were checked by another nuclear medicine physician with more than 10 years of experience in PET/CT diagnosis. The open-source imaging platform ITK-SNAP software (version 3.6; www.itksnap.org) was used to plot the ROIs of the corresponding lesions (17). The feature data were extracted, pre-processed, modeled, evaluated, and validated using the scikit-learn (sklearn, scikit-learn.org) packages in the python platform (18).

### Radiomics Feature Extraction

Data pre-processing: to ensure that the features were comparable, training/testing cohort division, missing-value filling, and data standardization were performed. First, to maintain the distribution of the original data, a stratified sampling method was applied to identify the training (501 samples, 70%) and testing cohorts (215 samples, 30%) (19). Moreover, the missing values (0 and 5) were filled with the median in the training and testing cohorts, respectively, and then, the same normalization was used for the data.

**Figure 1:**
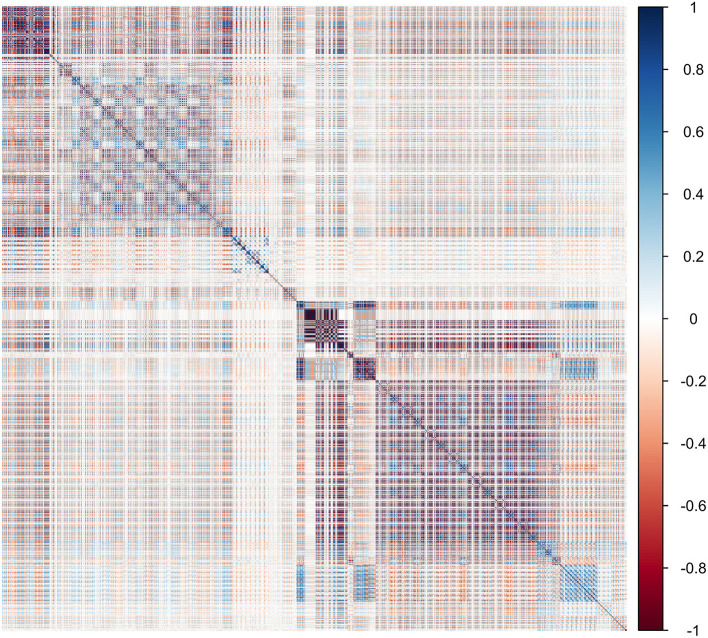
Heat map showing the correlation of radiomics features in the training cohort. The intensity of the relevance of each feature is displayed as a certain color. The darker the color, the higher the relevance, and the lighter the color, the lower the relevance.

There were 1,438 features of primary tumors that were automatically extracted using the sklearn packages. The Spearman rank order correlation coefficient was used to calculate the relationship between features, and the redundant features were eliminated with an average absolute correlation of 0.85 as the threshold. Support vector machine–recursive feature elimination, and the extremely randomized trees were applied to reduce the dimensions and select optimized features for the radiomics model (RM) to avoid the impact of redundant and unconnected features. The relevance of the association between each radiomics feature was established using heat maps (Figure 1). Consequently, a total of 25 principal correlative features, obtained through dimension reduction, were identified for inclusion in the RM to distinguish pN0 from pN1 and pN2. The results of the feature selection are shown in Table 1.

**Table 1:** The list of selected radiomics features.

**Characteristic type**	**Description**	**Selected features**
Histogram feature	Histogram parameters are related to the properties of individual pixels. They describe the distribution of voxel intensities within the images through the commonly used and basic metrics. Let X denote the 3D image matrix with voxels and the first-order histogram divided by discrete intensity levels.	PET_original_firstorder_Minimum
Textural phenotype features	Texture is one of the important characteristics used in identifying objects or regions of interest in an image. Texture represents the appearance of the surface and how its elements are distributed. It is considered an important concept in machine vision; in a sense, it assists in predicting the feeling of the surface (e.g., smoothness, coarseness, etc.) from image.	PET_textural_phenotype_level_H
Intra-peri-nodular textural transition features	Intra-peri-nodular textural transition features represents a minimal set of quantitative measurements which attempt to capture the transitional heterogeneity from the intra- to the peri-nodular space.	PET_Ipris_shell0_ge_mean
Partial local pattern binary feature	Partial local pattern binary feature is a local descriptor of the image based on the neighborhood for any given pixel. The neighborhood of a pixel is given in the form of P number of neighbors within a radius of R.	PET_PLBP_hist_tumor_orient6_0
		CT_PLBP_hist_tumor_orient2_7
		CT_PLBP_hist_tumor_orient2_3
		PET_PLBP_hist_tumor_orient3_1
		PET_PLBP_hist_tumor_orient4_3
		CT_PLBP_hist_tumor_orient1_2
High order texture feature based on wavelet transform	By using a family of functions localized in terms of time and frequency, wavelet transforms can centralize the energy of the original image within only a few coefficients after wavelet decomposition. These coefficients have high local relativity in three directions of different sub-band images: horizontal, vertical, and diagonal.	CT_wavelet-LHL_lbp-3D-m2_firstorder_90Percentile
		CT_wavelet-LLL_lbp-3D-m2_firstorder_InterquartileRange
		PET_wavelet-HLL_lbp-3D-m2_firstorder_Median
		PET_wavelet-HHL_lbp-3D-m1_firstorder_Skewness
		CT_wavelet-LHH_lbp-3D-m1_firstorder_Median
		PET_wavelet-LHL_lbp-3D-m1_firstorder_Median
		CT_wavelet-HLL_lbp-3D-m1_firstorder_90Percentile
		CT_WL_lbp_hist_cH1_1
		PET_WL_lbp_hist_cD1_4
		PET_wavelet-HLL_lbp-3D-m1_firstorder_Median
		CT_wavelet-HLL_lbp-3D-m2_firstorder_Range
		PET_wavelet-HHL_lbp-3D-k_firstorder_Minimum
		PET_WL_lbp_hist_cH2_2
		CT_wavelet-LLL_lbp-3D-m1_firstorder_Median
		CT_WL_lbp_hist_cV2_7

### Radiomics Modeling and Evaluation

**Figure 2:**
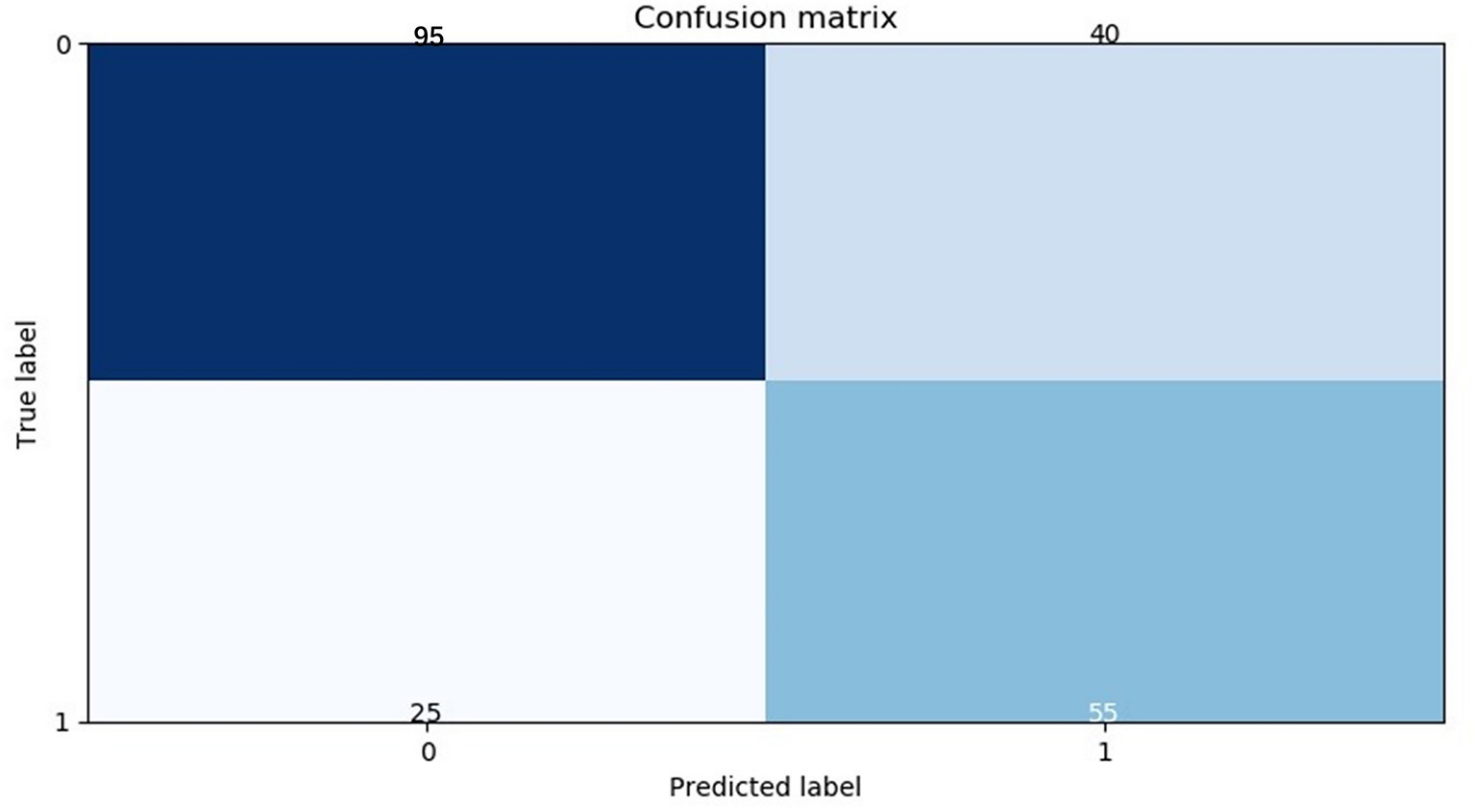
Confusion matrix of the radiomics model in the testing cohort. The abscissas and ordinates represent the true and predictive labels, respectively.

Extremely randomized trees was used as a classifier to model and optimize the radiomics signature in the modeling process. The 25 selected features were put into the classifier to build the RM to predict the pathological status of mediastinal LNs in the training cohort. Thereafter, a five-fold cross-validation of the training cohort was used to identify differences in the results. The training model was applied to the testing cohort for model validation. The area under the curve (AUC) of the receiver operating characteristic (ROC) curve was used as a means of quantitatively identifying the effective performance of the RM. The confusion matrix in the testing cohort was calculated (Figure 2).

### Statistical Analysis

Statistical analyses were conducted using the SPSS software, version 23.0 (IBM Corm., Armonk, NY), and *P*-values < 0.05 were deemed statistically significant.

The predictive abilities of the RM and cN were investigated using ROC analysis. The statistical significance of the improvement in the AUC after adding an explanatory factor was evaluated using the Delong test (20).

The clinicopathologic characteristics of the patients with a pN0 status were compared with those of the patients with pN1 and pN2. The training and testing data cohorts were compared to identify factors contributing to nodal metastasis using the χ^2^-test for categorical data and the one-sample *t-*test for continuous variables.

## Results

### Characteristics of All Patients

**Figure 3:**
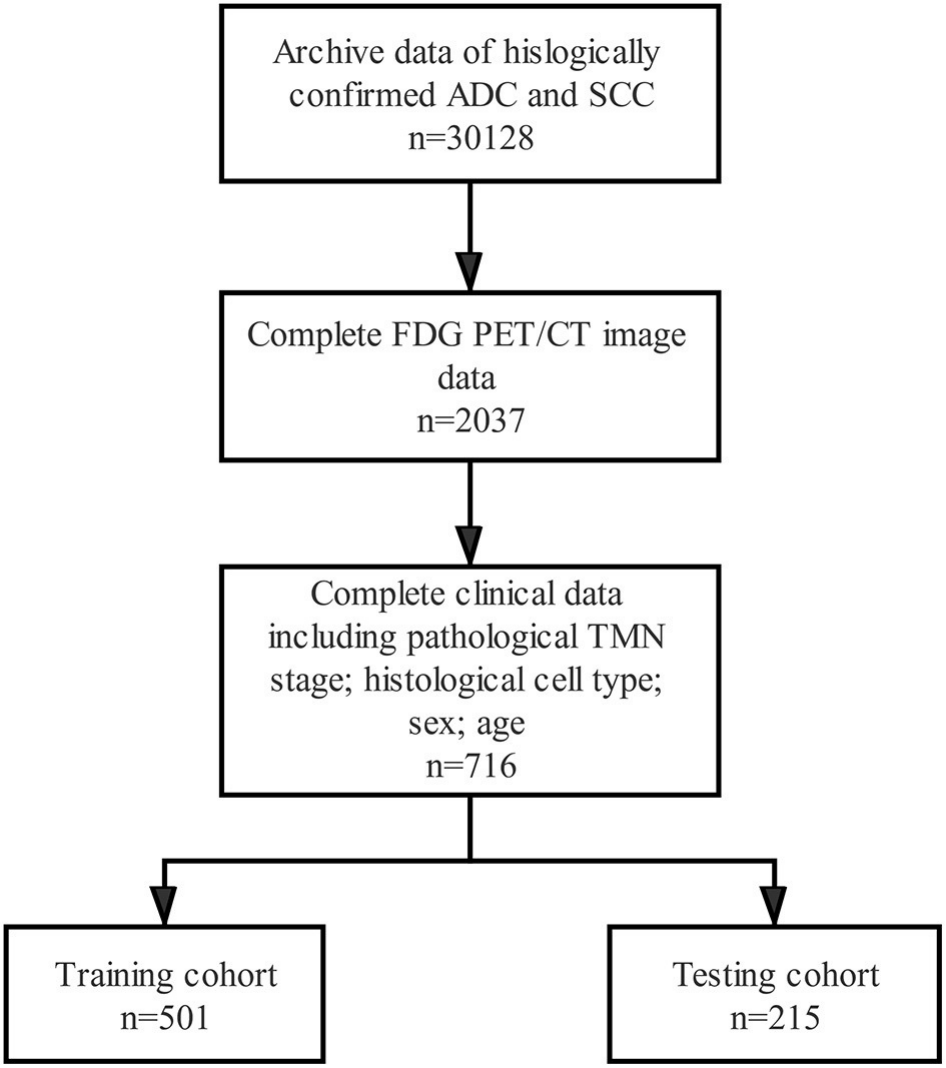
Flow diagram shows patient selection details.

The clinicopathological characteristics of the 716 patients enrolled in the study are shown in Table 2. Figure 3 shows the patient recruitment pathway. Among them, 220 were female and 496 were male, with an age range from 25 to 78 years. ADC was the most common histological type of NSCLC (417/716). The number of patients with SCC was 329. In the training cohort, the number of patients with pN0, pN1, and pN2 were 315, 74, and 112, respectively. In the testing cohort, the number of patients with pN0, pN1, and pN2 were 135, 43, and 37, respectively. The age of the training cohort, sex of the testing cohort, and anatomical classification of the two cohorts were statistically significantly different between the pN0 status and pN1 and pN2 status (*P* < 0.050). However, no significant difference was observed in the age of the testing cohort and in the sex of the training cohort (*P* > 0.050). Moreover, smoking history, lobar distribution, and histologic cell type were not significantly different between the two cohorts.

**Table 2:** Clinical characteristics.

**Characteristic**	**Training cohort**		***P***	**Testing cohort**		***P***
	**pN0 (*n* = 315)**	**pN1&2 (*n* = 186)**		**pN0 (*n* = 135)**	**pN1&2 (*n* = 80)**	
Age, mean ± SD, years	60 ± 9	58 ± 9	0.005^a^	60 ± 9	58 ± 9	0.152^a^
Gender, No. (%)			0.627			0.041
Male	212	128		90	66	
Female	103	58		45	14	
Smoking history			0.810			0.206
Yes	181	108		78	52	
No	134	78		57	28	
Lobar distribution			0.214			0.385
LUL	77	45		40	25	
LLL	46	28		25	15	
RUL	57	52		17	21	
RML	28	13		11	1	
RLL	107	48		42	18	
Anatomical classification			0.02			0.008
Central lung cancer	53	52		25	27	
Peripheral lung cancer	263	133		110	53	
Histologic cell type			0.696			0.697
SCC	109	67		52	38	
ADC	207	119		83	42	

*RUL, right upper lobe; RML, right middle lobe; RLL, right lower lobe; LUL, left upper lobe; LLL, left lower lobe.*

a *one sample T-test*.

### RM Performance

The diagnostic efficiency of the RM and cN were evaluated by the ROC curve. The AUC of the RM [0.81 (95% CI, 0.771–0.848); sensitivity: 0.794; specificity: 0.704] for the predictive performance of the pathological node status was significantly better than that of the cN in the training cohort [0.685 (95% CI, 0.644–0.728); sensitivity: 0.804; specificity: 0.568], (*P* = 8.29e-07, as assessed using the Delong test).

In the testing cohort, the AUC of the RM [0.766 (95% CI, 0.702–0.830); sensitivity: 0.688; specificity: 0.704] was also significantly higher than that of the cN [0.685 (95% CI, 0.619–0.747); sensitivity: 0.799; specificity: 0.568], (*P* = 0.0371, as assessed using the Delong test).

The above-mentioned nuclear medicine physicians excluded the patients with LNs significant enlargement and intense ^18^F-FDG uptake in PET/CT, and confirmed N1 or N2 by pathology from the whole population. The remaining 634 patients were defined as the cN ± group. Then, the sensitivity, specificity, and AUC of the cN and the RM in the cN ± group were calculated, respectively.

**Figure 4:**
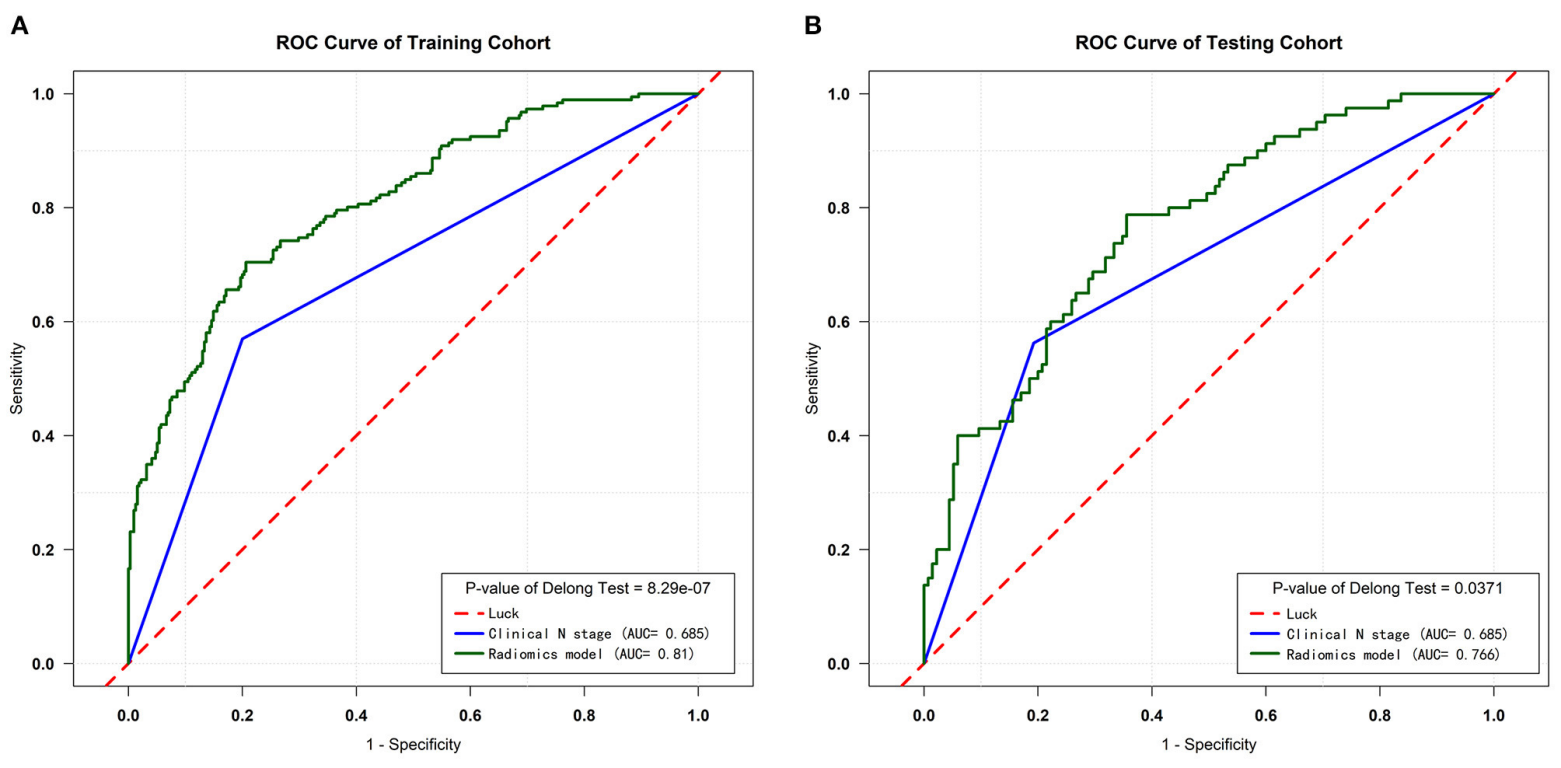
ROC curves of the training and testing cohorts. **(A)** ROC curves for the RM and cN of the training cohort. **(B)** ROC curves for the RM and cN of the testing cohort.

**Figure 5:**
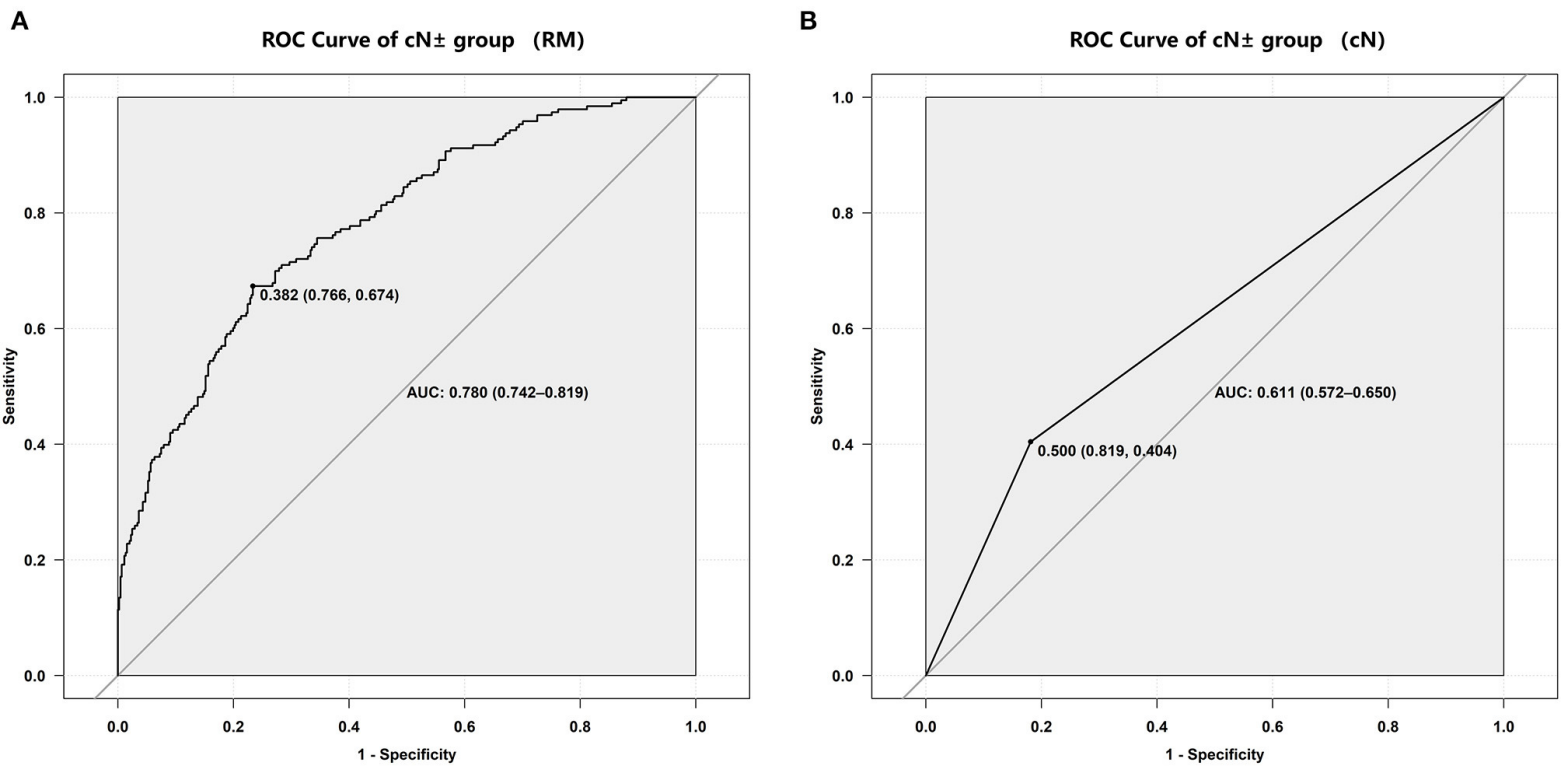
ROC curves of the cN ± group. **(A)** ROC curve for the RM. **(B)** ROC curve for the cN.

**Figure 6:**
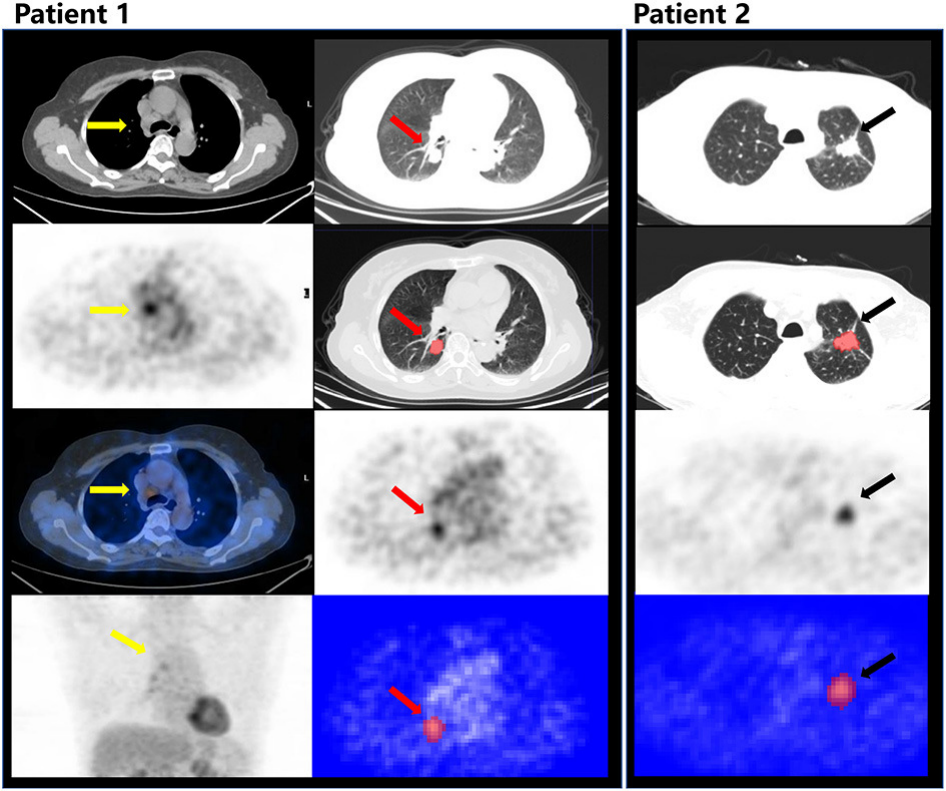
Patient 1: male, 55 years old, ADC, PET/CT showed lymph nodes in 4R (yellow arrow) with an intense FDG uptake, which was evaluated as cN2, predicted as N0 by the RM, and confirmed as pN0 after radical resection. The red arrow indicates the tumor in the right lower lobe (CT, PET) and the segmentation on ITK-SNAP. Patient 2: male, 66 years old, ADC, evaluated as cN0, but predicted as N+ by the RM, and confirmed as pN1 after radical resection. The black arrow indicates the primary lesion in the left upper lobe (CT, PET) and the segmentation on ITK-SNAP.

The RM showed the AUC of 0.802 (95%CI, 0.683–0.921) for the prediction of mediastinal LNs malignancy using the optimum cutoff value of 0.382 in the cN ± group. The sensitivity and specificity of the RM were 0.718 and 0.767, respectively. In comparison, the cN showed the AUC of 0.611 (95%CI, 0.572–0.650) using the optimum cutoff value of 0.500. The sensitivity and specificity of the cN were 0.819 and 0.404, respectively. The results demonstrated that the performance of the RM was effective in discriminating pN0 from pN1 and pN2 using the ^18^F-FDG PET/CT images. The performances of the RM and cN in the training and testing cohorts and the cN ± group are displayed in detail in Figures 4, 5, respectively, and the representative cases are presented in Figure 6.

## Discussion

In cases of NSCLC with a chance of cure, the standard surgical procedure is a pulmonary lobectomy with systemic mediastinal nodal dissection. However, some patients are not eligible for this therapy because of their advanced age or the presence of severe medical diseases, and some patients refuse surgical treatment. Limited surgery (wedge resection or segmentectomy) or SBRT would be alternatives for such patients. Sublobar resection helps preserve more healthy lung tissue, shortens the operative time, and improves the post-operative quality of life. Perioperative mortality and operative complication morbidity do not differ significantly between lobar and sublobar resection (3). SBRT has emerged as the preferred management strategy for patients who are not surgical candidates; however, for the selection of SBRT or restrictive surgery, accurate prediction of a pathological LN-negative status is a pre-requisite.

The diagnosis of NSCLC mediastinal LN metastasis is generally based on several parameters such as metabolism, size, morphology, and attenuation, which leads to dependence on clinical experience. In other words, the traditional practice involves treating medical images as pictures intended solely for visual interpretation (21). False-positive findings of mediastinal LNs are not uncommon in functional imaging with ^18^F-FDG PET/CT because the modality can mistakenly identify inflammation in patients with NSCLC due to infection, inflammation, or granulomatous diseases (8–10). The main molecular and pathological mechanisms of an avid FDG uptake in benign mediastinal LNs are lymphoid follicular hyperplasia and histiocyte infiltration associated with glucose transporter-1 overexpression (22). When benign mediastinal LNs manifest as a false-positive finding on PET imaging, and the CT morphology is not informative enough to support a judgment, there is an increased risk of an incorrect diagnosis. Benign high-uptake LNs can coexist with occult metastasis, making an accurate cN staging more difficult. In the clinical practice of mediastinal LN staging in NSCLC, nuclear medicine physicians are faced with the challenge of suspected positive LNs and possible OLM almost every day, which is difficult to deal with by relying solely on experience.

In the existing studies, for the accuracy of the cN staging in NSCLC, radiologists and nuclear physicians often analyzed parameters such as morphology and glucose metabolism or radiomic features of visible mediastinal LNs to improve the diagnostic ability of ^18^F-FDG PET/CT for metastasis (23–25). Gao et al. researched the method and efficacy of support vector machine classifiers based on texture features and a multi-resolution histogram to evaluate mediastinal LNs (11). Flechsig et al. used density as a threshold for the detection of malignant LN infiltration in a radiomics analysis of patients with NSCLC (12). Likewise, Lee et al.'s research indicated that the risk of mediastinal LN metastasis in NSCLC patients could be further stratified using both ^18^F-FDG uptake and LN density (24). Cho et al. attempted to determine the optimal cut-off values of the mediastinal LN standardized uptake values (SUV-LN)/primary tumor SUV (SUV-T) ratio to discriminate metastatic LNs from benign LNs (26). However, these researchers faced the common problem of an unpredictable OLM. Some algorithms for the analysis of parameters based on ^18^F-FDG uptake have been proposed in light of these limitations. Ouyang et al. used the primary tumor-to-blood SUV ratio and metabolic parameters in clinical N0 lung ADC to predict OLM (14). Kim et al. investigated the OLM's predictability using SUV, metabolic tumor volume (MTV), and total lesion glycolysis (TLG) in patients with cN0 lung SCC before surgery (15). There are no current studies of ^18^F-FDG PET/CT primary tumor-based radiomics classifiers of the LN staging (N0 vs. N1 and N2) in NSCLC. Therefore, if our solution proves to be feasible, it can be used to either differentiate benign and malignant LNs or determine OLM, thus leading to an informed therapeutic decision-making in the face of the challenge of false-positive and false-negative images.

Huang et al. have developed and validated a radiomics nomogram based on the primary tumor in a contrast-enhanced CT for pre-operative LN metastasis prediction in colorectal cancer (27). Inspired by their research achievement, we aim to introduce the radiomics modeling approach based on the ^18^F-FDG PET-CT images of the primary lesion into the LN staging in NSCLC. On the basis of the radiomics hypothesis, intratumoral heterogeneity detected by imaging could be the expression of genomic heterogeneity, which implies a worse prognosis because tumors with more genomic heterogeneity are more likely to be resistant to treatment metastasis (28). Mediastinal LN staging in NSCLC is highly correlated with prognosis; therefore, we assumed that LN metastasis information may be obtained from intratumoral heterogeneity. Some studies had discovered pre-therapy ^18^F-FDG PET/CT or CT-based radiomics classifiers of survival or response in patients with NSCLC (29–32). Therefore, it can obtain information from the primary lesion that is helpful for diagnosis or prognosis.

Nevertheless, the present study has several limitations too. Due to its retrospective design and performance at a single center, there is a risk of selection bias. A larger, multi-institutional prospective randomized study is needed to confirm these results.

## Conclusions

A Radiomics Model based on the ^18^F-FDG PET/CT analysis provided useful information for mediastinal LN staging in patients with NSCLC. Therefore, therapeutic planning could be tailored according to predictions, and limited surgery or SBRT could be helpful in patients with cN0.

## Data Availability Statement

The original contributions presented in the study are included in the article/supplementary material, further inquiries can be directed to the corresponding author.

## Ethics Statement

The studies involving human participants were reviewed and approved by Ethical Commission of Medical Research Involving Human Subjects at Region of Xiangya Hospital, Central South University, China. Written informed consent for participation was not required for this study in accordance with the national legislation and the institutional requirements. Written informed consent was not obtained from the individual(s) for the publication of any potentially identifiable images or data included in this article.

## Author Contributions

KZ designed the method, acquired the data, and wrote and edited the article. SH designed the method and approved the manuscript. Image analysis was performed by XW. Statistical analysis was performed by XW and CJ. YT, ZF, JH, and ZZ aided in data acquisition and interpretation. All authors contributed to the article and approved the submitted version.

## Conflict of Interest

XW was employed by the company GE healthcare (China). The remaining authors declare that the research was conducted in the absence of any commercial or financial relationships that could be construed as a potential conflict of interest.
